# The effect of intermittent drying on the cracking ratio of soybeans (*Glycine max*) at different relative humidity using reaction engineering approach modeling

**DOI:** 10.1002/fsn3.709

**Published:** 2018-06-28

**Authors:** Hwabin Jung, Won Byong Yoon

**Affiliations:** ^1^ Department of Food Science and Biotechnology College of Agriculture and Life Sciences Kangwon National University Gangwon Korea

**Keywords:** cracking, intermittent drying, model, reaction engineering approach, soybeans

## Abstract

Intermittent drying (ID) was applied to reduce soybean cracking because of the low moisture gradient and little thermal stress on soybeans during their tempering period. The drying temperature and relative humidity (RH) for the drying and tempering periods were 35°C and 20% and 25°C and 43%, respectively. The intermittency (*α*) of the drying was defined as the ratio of the drying period to the duration of the drying and tempering periods, and it varied at *α* = 1, 0.5, 0.4, and 0.25 to evaluate the drying characteristics and the soybeans’ quality. Intermittency processes redistributed the moisture in the soybean so that the low thermal stress was applied to the soybeans. The percentage of cracked grains increased with increasing the duration of drying period and decreasing tempering period. The moisture content and temperature changes during drying of soybeans were well fitted by reaction engineering approach (REA) modeling. Additionally, the physics that describe the soybeans’ drying behavior during ID were explained by the model parameters obtained from the REA modeling, such as the surface relative humidity and the surface water vapor concentration. ID showed the highest drying efficiency at *α* = 0.25 regarding the total drying time (13,800 s, i.e., the shortest drying time) and the lowest cracking ratio (<2.18%).

## 1 INTRODUCTION

Soybean is a well‐known valuable resource not only because of its healthy nutrients, such as high‐quality protein, oil, and phytochemicals, but also for its wide application in processed foods like tofu, soybean sauce, and soy milks (Prachayawarakorn, Prachayawasin, & Soponronnarit, 2006; Wardhani, Vázquez, & Pandiella, 2008). During the past decades, there has been an increasing soybean demand due to its use in the animal feed and human food industry (Dondee, Meeso, Soponronnarit, & Siriamornpun, 2011). Soybeans generally need to be dried for convenient storage, delivery, and processing. In addition, dried products are highly efficient for changing the ingredients into a powder form. The dehydration of soybeans increases their shelf life by controlling the water activity and the rate of chemical reactions associated with water. Nevertheless, the thermal cracking of soybean grain during drying reduces the germination value, seedling vigor, insect infestation, as well as the oil yield, and its quality (List et al., 1977; Liu, Haghighi, Stroshine, & Ting, 1990; White, Loewer, Ross, & Egli, 1976).

Hot‐air‐drying is the most common drying method. However, this procedure may cause a quality degradation of soybeans associated with rapid structural changes on the beans’ surface. In particular, the higher the temperature (>40.5°C) and the lower the humidity (<35%), the more the quality degradations occurred in soybeans (Mensah, Nelson, Hamdy, & Richard, 1985; Ting, White, Ross, & Loewer, 1980). A number of studies on the improvement of the drying efficiency and the dried soybeans’ quality have been conducted, such as the combination of near‐infrared radiation with fluidized‐bed drying (Dondee et al., 2011) and microwave drying (Wang, Li, Li, & Ye, 2009). In recent years, a time‐dependent drying method with an intermittency of the heat flux, obtainable by controlling the drying conditions, including various air temperatures, flow rates, or humidities, has been applied in food drying (Kumar, Karim, & Joardder, 2014). In particular, when drying heat‐sensitive grains such as soybeans, an intermittent drying (ID) method, that is, drying alternately with nondrying (tempering) periods, has been used for allowing time to redistribute the moisture from the internal to the external surface of the grain (Nishiyama, Cao, & Li, 2006). Several studies have demonstrated that ID improved the quality, functional chemical components, and energy efficiency during the drying process of rice, bananas, cocoa, and other food products (Aquerreta, Iguaz, Arroqui, & Virseda, 2007; Baini & Langrish, 2007; Chua, Mujumdar, & Chou, 2003; Hii, Law, & Cloke, 2009).

Developing mathematical models of the drying process is important to understand the drying behavior, to evaluate the drying performance, and to optimize the drying conditions (Putranto, Chen, Xiao, & Webley, 2011). Several drying models have been empirically or analytically developed. Empirical models possess the advantages of simple formulations and solutions, but they cannot describe the physics of drying which analytical models can (Baini & Langrish, 2007; Dhanushkodi, Wilson, & Sudhakar, 2017; Hii et al., 2009). However, analytical models are time‐consuming in solving partial differential equations associated with the complex drying phenomena of agricultural products or food (Esfahani, Majdi, & Barati, 2014; Irigoyen & Giner, 2017; Silva, Precker, Silva, & Gomes, 2010). The reaction engineering approach (REA) was developed, based on the assumption that drying is a competitive process between an “evaporation reaction” and a “condensation reaction” (Chen & Xie, 1997). The REA model has been suggested as accurate, simple, and practical for both conventional continuous drying and ID (Chen, Pirini, & Ozilgen, 2001; Putranto, Chen, Devahastin, Xiao, & Webley, 2011; Putranto, Xiao, Chen, & Webley, 2011; Putranto, Chen, Xiao, et al., 2011).

Although semi‐empirical approaches with thin layer models (Rafiee et al., 2009), numerical simulations using the finite element methods (Rafiee, Keyhani, & Mohammadi, 2008), analytical solutions with coupled mathematical models (Hemis & Raghavan, 2014), and fractional‐order kinetic models (Nicolin, Defendi, Rossoni, & de Matos Jorge, 2017) have been conducted to describe soybean drying characteristics at varied drying temperature and relative humidity, neither of them modeled the time‐varying drying process to improve the quality of soybeans. Moreover, the simple and accurate REA model has not been used to evaluate the drying process of soybeans. Therefore, the aims of this study were (a) to investigate the efficiency of the ID method on soybean drying compared with conventional hot‐air‐drying, (b) to determine the drying characteristics of soybeans using REA, and (c) to estimate the effect of intermittency during ID on the soybean quality after applying each drying process.

## 2 MATERIALS AND METHODS

### 2.1 Sample

The soybeans (*Glycine max*) used in this study were provided by the National Institute of Crop Science (NICS, Miryang, Gyeongnam Province, Republic of Korea), harvested directly from the field, and stored at 4°C with 80% RH. The moisture content of soybean was obtained by heating in an oven at 105°C for 24 hr (AOAC, 1995). The initial moisture content of the soybeans was 0.25 ± 0.003 kg/kg (d.b.) or 20 ± 0.6% (w.b.).

### 2.2 Soybean drying

Continuous drying and ID were conducted in a convective dryer (NB‐901M; N‐BIOTEK Inc., Bucheon, Gyeonggi, Republic of Korea). The soybean has an ellipsoidal shape with three axes of different length. All axes of 20 soybeans were measured using a vernier caliper during the drying process to determine the change in the soybeans’ average length (*L*) and surface area (*A*). The relationships among the moisture content, the average length, and the surface area were assessed by a linear regression analysis (*R* ^2^ > 0.98). Soybeans of approximately 30 g were placed on a meshed plastic tray, forming a single layer with the initial temperature of 25 ± 1°C. The center and surface temperatures inside soybeans were measured using a fiber‐optic temperature sensor (MultiSens, Opsens Solutions, Quebec, Canada). The temperature and humidity controls at 35 ± 1°C and 20 ± 1% were obtained using a dryer with a convective airflow of 3 m/s. The tempering was conducted by placing a tray with soybeans outside of the dryer. The room temperature and humidity were maintained at 25 ± 1°C and 43 ± 2%, respectively. For analysis of the moisture, the sample's weight change was recorded using a digital scale having an online measuring and data collection system (AX8201; Ohaus Scale Corp, Florham Park, NJ, USA) with an accuracy of 0.01 g during drying. The measured equilibrium moisture content of the soybeans (*X*_*b*_) was determined at the end of drying for 82 hr.

Intermittent drying was conducted by 600, 1,200, and 1,800 s drying, followed by tempering for 1,800 s. The constant tempering time (i.e., 1,800 s) was applied to evaluate the effect of the intermittency (*α*) defined as the ratio of the drying period to the duration of the drying and heating period:

(1) $$\alpha =\frac{t_{D}}{t_{D}+t_{T}}$$

where *t*_*D*_ is the drying time period's duration (s) and *t*_*T*_ is the tempering time period's duration (s) of each cycle.

### 2.3 Soybean cracking

The soybean cracking was visually sorted out with a scale and fluorescent light after the moisture content reached 0.134 ± 0.001 kg/kg (d.b.). The cracking percentage (*C* [%]) was calculated using the following equation:

(2) $$C\left(\%\right)=\frac{S_{C}}{S_{T}}\times 100$$

where *S*_*C*_ and *S*_*T*_ are the number of cracked soybean grains and the number of total soybean grains, respectively.

### 2.4 Mathematical modeling of soybeans using the reaction engineering approach

The REA is a semi‐empirical model developed based on the concept of a chemical reaction that predicts the complicated nature of food material using a simple lumped parameter. The REA model in drying presumes that moisture has to overcome an energy barrier to be evaporated, whereas condensation does not. The activation energy for the removal of moisture in this process is related to the moisture content of the sample and used as the “fingerprint” of the individual material (Chen & Xie, 1997).

The evaporation rate of moisture at the interface of the sample can be expressed as follows:

(3) $$m_{s}\frac{\mathrm{dX}}{\mathrm{dt}}=-h_{m}A\left(\rho_{v,s}-\rho_{v,b}\right)$$

where *m*_*s*_ is the dried mass of the sample (kg), *X* is the moisture content of the sample (kg/kg), ρ_*v,s*_ is the vapor concentration of the sample at the interface between the sample and drying air (kg/m^3^), ρ_*v,b*_ is the vapor concentration of drying air (kg/m^3^), *h*_*m*_ is the mass transfer coefficient (m/s), *A* is the surface area of the sample (m^2^), and *t* is time (s).

The surface vapor concentration (ρ_*v,s*_), which is a time‐dependent variable and difficult to measure, can be calculated using a relationship to the surface's relative humidity as follows:

(4) $$\rho_{v,S}=RH_{S}\rho_{v,sat}\left(T_{S}\right)$$

where *RH*_*s*_ is the relative humidity at the sample surface and *T*_*s*_ is the temperature at the interface between the sample surface and drying air (K). The saturated vapor concentration (ρ_*v,sat*_) at the sample–air interface can be defined as a function of the surface temperature using original data obtained from previous studies (Incropera & DeWitt, 2002; Keey, 1992):

(5) $$\begin{array}{cc} \rho_{v,sat} & \;=\;4.844\times 10^{-9}{\left(T_{S}-273\right)}^{4}-1.4807\;\times \;10^{-7}{\left(T_{S}\;-\;273\right)}^{3}\;+2.6572\\ & \;\times \;10^{-5}{\left(T_{S}-273\right)}^{2}\;-\;4.8613\;\times \;10^{-5}\left(T_{S}\;-\;273\right)+\;8.342\;\times \;10^{-3} \end{array}$$

The correlation between ρ_*v,sat*_ and the temperature in Equation (5) was formulated using the data for 273.15 K < *T*_*s*_ < 430 K (Patel, Chen, Lin, & Adhikari, 2009).

The relative humidity at the surface (*RH*_*s*_) can be expressed by a variant of the Arrhenius equation (Equation (6)) with the introduction of an apparent activation energy term representing the difficulty in the moisture's removal at different drying stages and a change in this value with the moisture content could reflect structure changes (Chen, 2008; Chen & Xie, 1997):

(6) $$RH_{S}=\exp\left[-\frac{\Delta E_{v}}{RT_{S}}\right]$$

where ∆*E*_*v*_ is the apparent activation energy.

Using Equations (3), (4), and (6), the mass balance can be expressed as (Chen, 2008):

(7) $$m_{S}\frac{\mathrm{dX}}{\mathrm{dt}}=-h_{m}A\left[\exp\left(\frac{-\Delta E_{v}}{RT_{S}}\right)\rho_{v,sat}-\rho_{v,b}\right]$$

The activation energy (∆*E*_*v*_) can be calculated by Equation (8), which is a rearranged form of Equation (7) (Chen & Xie, 1997):

(8) $$\Delta E_{v}=-RT_{S}\ln\left[\frac{-m_{S}\frac{\mathrm{dX}}{\mathrm{dt}}\frac{1}{-h_{m}A}+\rho_{v,b}}{\rho_{v,sat}}\right]$$

where the parameters are experimentally determined.

The activation energy can be associated with the sample‐free moisture content and normalized using the maximum possible activation energy (∆*E*_*v,b*_), that is, the maximum ∆*E*_*v*_ at the equilibrium condition of the drying process. The relative activation energy (∆*E*_*R*_) versus free moisture content (*X − X*_*b*_) may characterize the inherent drying characteristics of individual material reacting to various drying conditions and reflects possible structure changes as the difficulty of removing moisture is involved in different drying stages (Chen, 2008). This relationship is obtained by a simple polynomial regression fitting.

(9) $$\Delta E_{R}=\frac{\Delta E_{v}}{\Delta E_{v,b}}=f\left(X-X_{b}\right)$$

where *X*_*b*_ is the equilibrium moisture content corresponding to the relative humidity and temperature of the drying air and ∆*E*_*v,b*_ is the equilibrium activation energy which can be calculated by substituting *RH*_*b*_ for *RH*_*s*_ and *T*_*b*_ for *T*_*s*_ in Equations (6) and (10) (Patel et al., 2009).

(10) $$\Delta E_{v,b}=-RT_{b}\ln\left(RH_{b}\right)$$

where

(11) $$RH_{b}=\frac{\rho_{v,b}\left(T_{b}\right)}{\rho_{v,sat}\left(T_{b}\right)}$$

The activation energy ratio in Equation (9) is called the relative activation energy. It is used in the mass balance of the REA to yield the activation energy (Δ*E*_*v*_) change during the drying process reflecting the difficulty to remove moisture from the material depending on the drying stage. Therefore, the relative activation energy curve can be extended by a single curve to various drying conditions such as intermittent drying.

For modeling the moisture and the temperature changes during the soybeans’ drying process, the mass balance of the REA (Equation (7)) must be coupled with the energy balance of the sample. The energy balance equation is as follows:

(12) $$\frac{d\left(mC_{p}T\right)}{\mathrm{dt}}\approx hA\left(T_{b}-T\right)+m_{S}\frac{\mathrm{dX}}{\mathrm{dt}}\Delta H_{v}$$

where *m* is the mass of the sample, *C*_*p*_ refers to the specific heat capacity of the sample, *h* is the heat transfer coefficient, and *∆H*_*v*_ is the latent heat of the moisture evaporation.

The heat and mass transfer coefficients *h* and *h*_*m*_ in Equations (7) and (12) were obtained with the Sherwood number (*Sh*) and the Nusselt number (*Nu*), respectively, following the research of Putranto, Chen, Xiao, et al. (2011) for drying coffee and rice:

(13) Sh=0.511Re0.5Sc0.37=hmLD

(14) Nu=0.511Re0.5Pr0.37=hLk

where *Re* is the Reynolds number, *Sc* is the Schmidt number, *Pr* is the Prandtl number, *L* is a characteristic length of the sample, *k* is the thermal conductivity of the drying air, and *D* is the diffusivity of the drying air.

The dimensionless numbers are defined by Equations (15), (16), (17), respectively.

(15) Re=ρuLμ

(16) $$Sc=\frac{\mu }{\rho D}$$

(17) $$\mathrm{Pr}=\frac{\mu C_{p,air}}{k}$$

where ρ is the density, *u* is the drying air's velocity, μ is the dynamic viscosity, and *C*_*p,air*_ is the specific heat of the drying air. The thermophysical and transport properties of the drying medium (air) were calculated using the correlations obtained from plotting the temperature and the property reported by Incropera and DeWitt (2002) (Patel et al., 2009).

The coupled differential equations containing the mass and energy balance equations (Equations (7) and (12)) were solved simultaneously using the MATLAB^®^ (R2015a; MathWorks, Natick, MA, USA) ODE solver ode23s. The prediction of a drying process is possible with both the known initial moisture content and drying conditions (drying air temperature and humidity) when solving the coupled differential equations while applying the relative activation energy (Δ*E*_*R*_) to the same material.

## 3 RESULTS AND DISCUSSION

### 3.1 The moisture content profile and soybean modeling during drying

The experimentally recorded data, such as the change in the soybeans’ moisture content $\left(m_{S}\frac{\mathrm{dX}}{\mathrm{dt}}\right)$, the sample temperature (*T*), the soybeans’ surface area (*A*), and the parameters along with the drying air temperature (*T*_*b*_) throughout the drying process, were used for calculating the relative activation energy (Equation (9)) which was obtained from Equation (8) and (10). An empirical equation of the relative activation energy (Δ*E*_*R*_) versus the moisture content difference (*X* − *X*_*b*_) from the measured parameters was obtained by a nonlinear regression analysis. The third‐order polynomial function described the relative activation energy's relationship for the drying well (*R* ^2^ > 0.99). The data were generated by continuous drying of soybeans at 35°C (*α* = 1).

The polynomial equation obtained from this relationship, used to implement mathematical models for various soybean drying schemes, was as follows:

(18) $$\begin{array}{cc} \Delta E_{R} & =-213.38{\left(X-X_{b}\right)}^{3}+38.831{\left(X-X_{b}\right)}^{2}\\ & -4.2124\left(X-X_{b}\right)+0.9958 \end{array}$$

**Figure 1:**
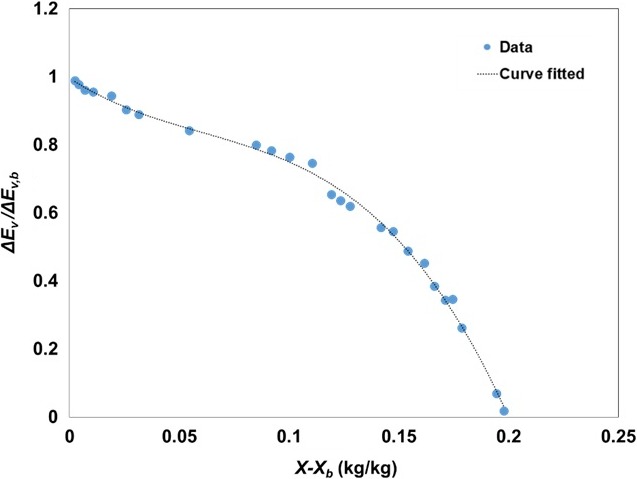
The relative activation energy curve of soybeans by free moisture content

The relative activation energy increased as the free moisture content decreased as shown in Figure 1. This indicates that more energy is required as a higher amount of pure water evaporates on the surface of the material. When the free moisture has completely evaporated from the material, the surface activation energy reaches the equilibrium activation energy (Lin & Chen, 2006).

**Figure 2:**
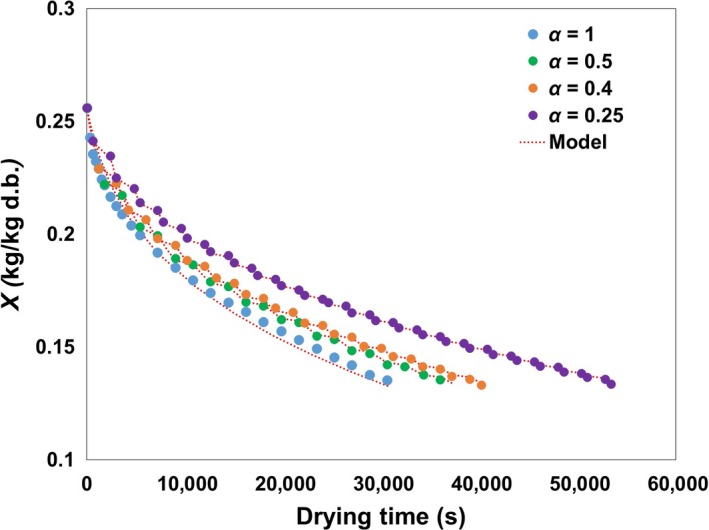
Moisture content profiles of soybeans’ continuous and intermittent drying with various intermittencies (*α* = 1, 0.5, 0.4, and 0.25)

The heat and mass balances (Equation (7) and (12)) are solved simultaneously to obtain the moisture and the temperature changes during ID with the relative activation energy (Equation (18)) representing an intrinsic drying behavior of soybeans for changing the moisture content and the sample temperature. The moisture changes during continuous drying and the ID of varying soybean intermittencies with the fitted model are shown in Figure 2.

There was a good agreement between the obtained results and the calculated REA model for both continuous drying and ID of soybeans with an *R* ^2^ greater than 0.99 and a RMSE lower than 0.4252 (Table 1). The relative activation energy (a lumped parameter representing a material's intrinsic drying property) of the REA could achieve accuracy under dynamic drying conditions because of its flexibility expressed as a function of the moisture content's difference. The normalized relative activation energy (Δ*E*_*R*_) using the equilibrium activation energy (Δ*E*_*v,b*_) may appropriately express the physics during intermittent drying as much as the general complex diffusion models (Putranto, Chen, Xiao, et al., 2011; Silva et al., 2010; Váquiro, Clemente, García‐Pérez, Mulet, & Bon, 2009).

**Table 1:** *R* ^*2*^ and *RMSE* of moisture content and temperature profiles predicted by REA model

Drying scheme	Moisture content		Temperature	
	*R* ^*2*^	RMSE	*R* ^*2*^	RMSE
*α* = 1	0.999	0.0496	0.999	0.4252
*α* = 0.5	0.999	0.0164	0.997	0.3699
*α* = 0.4	0.999	0.0120	0.996	0.3846
*α* = 0.25	0.999	0.0080	0.996	0.4083

The relative activation energy of the REA model reflects the degree of difficulty in drying that correlates well with the drying rate. In addition, the REA allows a natural transition from the constant drying rate period to the falling drying rate period, whereas other drying modeling approaches have to define the critical water content (Chen, 2008; Kowalski & Pawłowski, 2010). The REA model is related to the characteristic drying rate curve (CDRC) model. Both the REA and the CDRC models need an experimentally determined empirical curve, but the former possesses the parameters which can explain more physics of drying process than the CDRC model (Chen, 2008). Chen and Lin (2005) fitted the drying of skim milk and whole milk droplets to both models and determined that the REA model was more accurate. Putranto, Chen, Devahastin, et al. (2011) obtained a better result for the REA model than the modeling approach by Kowalski and Pawłowski (2010) with respect to the intermittent drying of kaolin. The REA modeling for time‐varying drying in studies of rice, mangoes, and polymer solutions also exhibited a good agreement with experimental data (Putranto, Chen, & Webley, 2010; Putranto, Chen, Xiao, et al., 2011).

By solving the REA model, the total drying times to reach the approximate target moisture content (0.134 ± 0.001 kg/kg [d.b.]) (i.e., assuring the shelf life of soybeans in the ambient temperature) at *α* = 1, 0.5, 0.4, and 0.25 were 30,600, 37,800, 40,200, and 53,400 s, respectively. The soybeans usually dried to the target moisture content of about 12% w.b. in this study which minimized a quality deterioration when stored.

However, the calculated approximate total drying time periods of each scheme, representing the dryer's total operating time, were 30,600, 19,800, 16,800, and 13,800 s, respectively. This demonstrated that the moisture migrates from the internal to the outward part of the grain during tempering to reduce the moisture gradient of the soybeans. It is well‐known that the redistribution of moisture and the temperature during ID contribute to an improvement in the energy efficiency (Kumar et al., 2014). Cao, Nishiyama, and Koide (2004) and Nishiyama et al. (2006) reported that tempering enhanced the drying rate of the later drying period compared with continuous drying of food materials. The surface moisture was initially removed with ease and lowered, due to the surface mass transfer from the samples to the drying medium (air). The tempering period increased the moisture at the surface by the moisture migration from the internal to external samples’ parts, which explains why the driving force for the mass transfer between the sample and the drying air is higher at a later drying stage in intermittent drying than in continuous drying.

### 3.2 Drying temperature modeling and the simulated drying parameters from REA modeling

**Figure 3:**
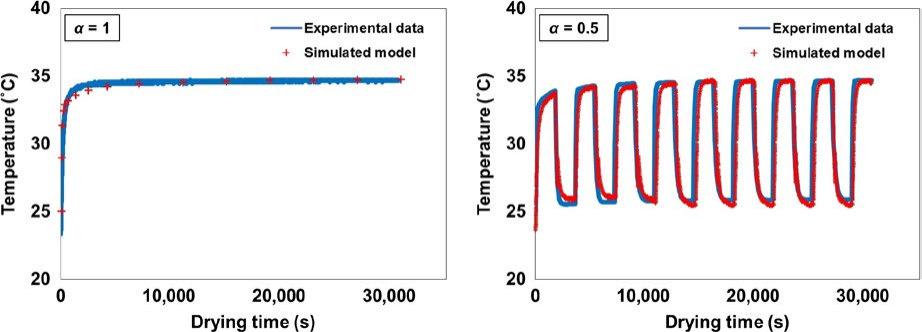
Temperature profiles of soybeans’ continuous (*α* = 1) and intermittent drying (*α* = 0.5)

**Figure 4:**
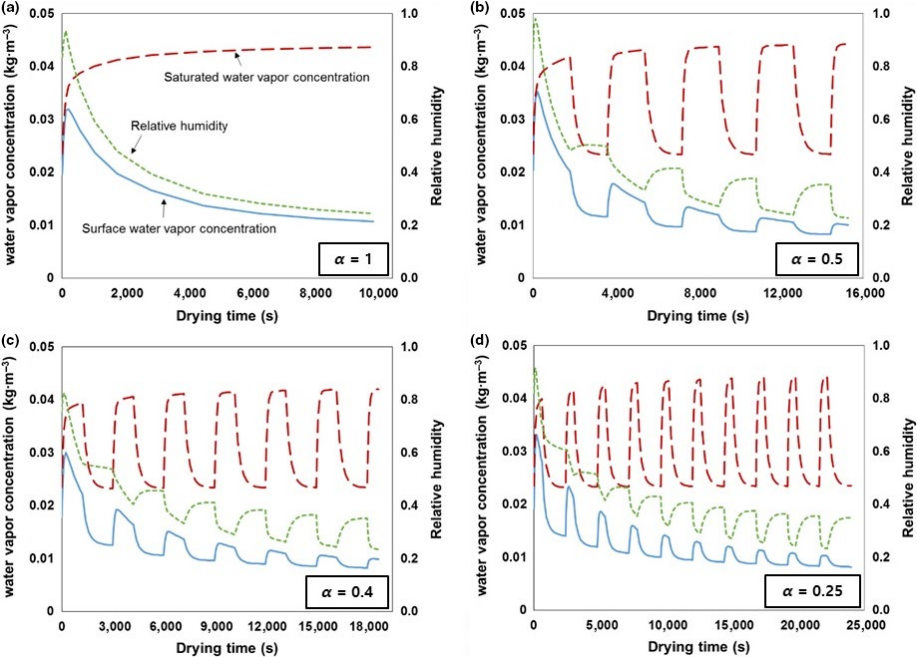
Water vapor concentrations and surface relative humidity profiles of the drying schemes

The temperature modeling results of soybean drying using the REA with various drying schemes are presented in Figure 3. The predicted temperature profiles were consistent with the experimental results (*R* ^2^ **> 0.99) which indicate that the REA can model and predict intermittent soybean drying with a high accuracy. The predictions of surface water vapor concentration, saturated water vapor concentration, and surface relative humidity at different *α* values are depicted in Figure 4. These REA model parameters help in the analysis, and the understanding of the mechanisms involved in continuous and intermittent drying (Chen, 2008; Putranto, Chen, Xiao, et al., 2011).

The relative humidity of the surface and the surface water vapor concentration for continuous soybean drying (*α* = 1) of the total drying time (about 30,800 s) decreased gradually from 0.032 kg/m^3^ and 0.95 to 0.011 kg/m^3^ and 0.27, respectively, whereas the saturated water vapor concentration increased from 0.024 to 0.044 kg/m^3^ because it depends on the soybean temperature (Figure 4a). The surface profiles of its water vapor concentration and relative humidity in ID displayed fluctuations with intermittencies. For the ID schemes (*α* = 0.5, 0.4, and 0.25) in Figure 4b–d, the saturated water vapor concentration increased rapidly during the first drying period for 1,800, 1,200, and 600 s due to heat from the drying air transferred to the soybeans. The surface's relative humidity and vapor concentration decreased during the drying period as a consequence of moisture transfer to the air (Putranto, Chen, Xiao, et al., 2011). After the drying period, the saturated water vapor concentration lowered with the soybeans’ temperature decrease by tempering.

The surface vapor concentration is influenced by both the surface's temperature and its relative humidity (Putranto et al., 2010). The evaporation of moisture from the soybean–air interface to the drying air leads to a low surface relative humidity and surface vapor concentration, while the surface relative humidity rises during the tempering period because the drying air temperature is lower than that of the soybeans and consequently, the moisture may accumulate on the soybeans’ surface (Putranto, Chen, Xiao, et al., 2011). In addition, both the surface relative humidity and the surface water vapor concentration were reduced at the latter's intermittency cycle because the moisture content of soybeans continues to decrease as drying proceeds. The maximum surface relative humidity and the surface vapor concentration for the ID schemes were 0.98 and 0.035 kg/m^3^ and decreased at the end of drying to 0.226–0.227 and 0.009 kg/m^3^, respectively.

When the soybean moisture content reached 0.134 ± 0.001 kg/kg (d.b.), the final surface water vapor concentration at the end of the drying period was 0.009 kg/m^3^ for all ID schemes despite their reduced total heating times, which were 19,800, 16,800, and 13,800 s for *α* = 0.5, 0.4, and 0.25, respectively. With respect to the latter part of ID, the effect of intermittency was lower when the surface's saturated water vapor concentration at the end of the drying period and the tempering period's beginning displayed only a small difference. Therefore, the parameters generated from REA modeling can help to determine the total number of the intermittency cycles and the total drying time for saving time and energy during the drying process (Putranto, Chen, Xiao, et al., 2011).

### 3.3 Effects of drying on cracking soybean grains

**Figure 5:**
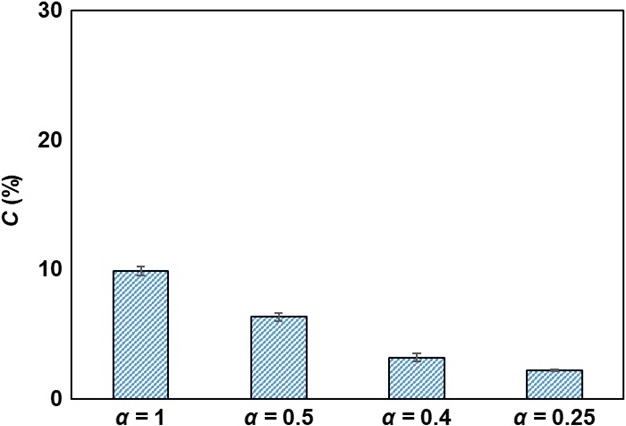
Percentage of cracked soybeans at varied intermittency schemes

The percentage of cracked soybeans after drying at varied intermittencies is shown in Figure 5. The results show that the cracking of soybeans was decreased with an intermittency decrease being 9.88 ± 0.34%, 6.32 ± 0.33%, 3.16 ± 0.31%, and 2.17 ± 0.07% for *α* = 1, 0.5, 0.4, and 0.25, respectively. It is readily apparent that the time of the drying period has a marked effect on soybean cracking. As shown in Figure 5, the percentage of cracked soybeans decreased as the frequency of the intermittency cycle rose when a tempering period was applied and the time of drying shortened as well. The profiles of the vapor concentrations and the surface relative humidity generated by REA modeling demonstrated that the decrease in the surface relative humidity diminished with temperature fluctuations (Figure 4b–d). In addition, the surface relative humidity with a low intermittency (*α*) retained high values as the drying period is shorter than the tempering period. It has been reported that a low temperature and a high relative humidity of drying air reduce seed coat cracks in soybeans (Walker & Barre, 1972). Conventional hot‐air‐drying methods such as batch drying or fluidized‐bed drying have drawbacks that cause serious damages to the physical qualities of soybean grains (40%–60% of damage) (Dondee et al., 2011; Soponronnarit, Swasdisevi, Wetchacama, & Wutiwiwatchai, 2001). Generally, cracking is inevitable because the drying process leads to a moisture gradient between the soybeans’ inside and their surface by high heat and mass transfer rates. However, a reduction in soybean cracking to less than 3% is possible in ID due to the moisture redistribution inside the soybeans during the tempering period. Aquerreta et al. (2007) and Dong, Lu, Liu, Koide, and Cao (2010) thus concluded that the moisture gradient's decrease in rice kernels during tempering notably lowered the proportion of fissured kernels when ID was performed. The simulated surface relative humidity of soybeans using REA modeling could depict the cracking ratio of soybeans during drying. Therefore, the moisture redistribution and the low thermal stress to the soybean grain during ID can also be explained by the surface's relative humidity which suggests that a reduction in the cracking of soybeans can be achieved by proper intermittency scheming.

## 4 CONCLUSION

The effects of the ID process with various intermittencies on soybeans were investigated. The results indicated that REA modeling fitted the drying kinetic of soybeans under time‐varying drying conditions well. The maintenance of soybean grains’ physical quality was achieved by controlling the intermittency. The lowest cracking ratio was below 2.18% with the most frequent intermittency, *α* = 0.25. Additionally, the tempering period's existence was beneficial in shortening the total dryer operation time by the redistribution of moisture. The REA can predict the surface water vapor concentration and the surface relative humidity, which is useful for the interpretation of drying phenomena during ID. Additionally, the REA model is instrumental in the identification of relationships between drying conditions and the soybean cracking ratio during drying. The REA model has been applied to predict changes in the moisture content and the temperature of soybeans in the process of intermittent drying. Furthermore, the physics of ID were explained with the parameters estimated by the REA model, such as the surface relative humidity (RH_s_) and the surface water vapor concentration (ρ_*v,s*_), which are generally difficult to measure experimentally.

## CONFLICT OF INTEREST

The authors declare no conflict of interests.
